# Supplementary material for: Wearable accelerometers reveal objective assessment of walking symmetry and regularity in idiopathic scoliosis patients
Source: PeerJ. 2024 Jul 16;12:e17739. doi: 10.7717/peerj.17739 (PMC11259127; doi:10.7717/peerj.17739)
Supplement: Supplemental Information 3 [file peerj-12-17739-s003.docx]

1、%% Using X-axis data as an example, filtering method, x_DOT3 as raw acceleration data

Wc = 2 * 10 / 60; % computes the normalized cutoff frequency for the Butterworth filter, with 10 Hz as the cutoff frequency and 60 Hz as the sampling frequency

[lags, rxx] = butter(2, Wc, 'low'); % Design a second-order Butterworth low pass filter for X-axis data

x_FilDOT3 = filtfilt(lags, rxx, x_DOT3); % Apply the filter to the x_DOT3 acceleration data

% Plot the original acceleration data for the X-axis

subplot(2, 1, 1);

plot(t_DOT3, x_DOT3, 'r', 'linewidth', 1.5);

title('Original Acceleration Data (x\_DOT3)');

% Plot the filtered acceleration data for the X-axis

subplot(2, 1, 2);

plot(t_DOT3, x_FilDOT3, 'r', 'linewidth', 1.5);

title('Filtered Acceleration Data using Butterworth Low-pass Filter (x\_FilDOT3)');

2、%% Using X-axis data as an example, calculating the Unbiased Autocorrelation Coefficient Sequence

[rxx_dot3, lags] = xcov(x_FilDOT3, x_FilDOT3, 'unbiased'); % X-axis

rxx_dot3 = rxx_dot3 - mean(rxx_dot3); % This line removes the mean from the autocorrelation sequence to center it around zero

% Normalize the coefficients to 1.0 at zero lag

rxx_dot3 = rxx_dot3 / rxx_dot3((numel(rxx_dot3) + 1) / 2);

% Plot the unbiased autocorrelation coefficient sequence

figure;

subplot(311); plot(lags, rxx_dot3);

xlim([-300, 300]); % The window length is set at a constant 5 s, 5 * 60 Hz

xlabel('Lags\_dot3');

ylabel('Unbiased autocorrelation coefficient');

title('X-axis');

grid on;

3、 % Calculate gait harmonic ratio in AP (X), VT (Z), ML (Y) directions, acceleration data is accx, accz, accy

% Calculate the number of DOT3_HR

startx_DOT3 = 43; % Starting position

peri = 31; % Gait period value from Figure 2 (D1)

numHRs_DOT3 = floor((420 - startx_DOT3 + 1) / (peri * 2)); % 420 is the total number of data points, calculating the number of HRs

% Initialize arrays to store each HR

HRs_AP_d3 = zeros(1, numHRs_DOT3);

HRs_ML_d3 = zeros(1, numHRs_DOT3);

HRs_VT_d3 = zeros(1, numHRs_DOT3);

% Calculate the harmonic ratio for each HR

% Set the range for the gait period

startRow = startx_DOT3;

endRow = startRow + (peri * 2);

for i = 1:numHRs_DOT3

% Calculate data within the current gait period

accxD3 = x_FilDOT3(startRow:endRow, :);

accyD3 = y_FilDOT3(startRow:endRow, :);

acczD3 = z_FilDOT3(startRow:endRow, :);

% Check data length consistency

if length(accxD3) ~= length(acczD3) || length(accxD3) ~= length(accyD3)

error('Input data lengths are inconsistent. Please ensure the lengths of acceleration data in three directions are the same.');

end

Fs = 60; % Hz, sampling frequency

N_d3 = length(acczD3); % Signal length

f = Fs * (0:N_d3/2-1) / N_d3; % Frequency vector

y_APd3 = fft(accxD3) / N_d3; % Perform Fourier transform

y_VTd3 = fft(acczD3) / N_d3; % Perform Fourier transform

y_MLd3 = fft(accyD3) / N_d3; % Perform Fourier transform

% Calculate single-sided amplitude spectrum

NN_d3 = round(N_d3 / 2);

xa_w3 = abs(y_APd3(1:NN_d3 + 1, :));

za_w3 = abs(y_VTd3(1:NN_d3 + 1, :));

ya_w3 = abs(y_MLd3(1:NN_d3 + 1, :));

% Remove DC component

xa_d3 = xa_w3(2:end, :);

ya_d3 = ya_w3(2:end, :);

za_d3 = za_w3(2:end, :);

% Extract amplitudes of the first 20 harmonics

harmonics = 20;

if length(xa_d3) < harmonics || length(ya_d3) < harmonics || length(za_d3) < harmonics

error('Number of harmonics exceeds signal length. Please check gait period and data length.');

end

harmonics_3x = xa_d3(1:harmonics);

harmonics_3y = ya_d3(1:harmonics);

harmonics_3z = za_d3(1:harmonics);

% Calculate gait harmonic ratio

HR_AP_d3 = sum(harmonics_3x(2:2:end)) / sum(harmonics_3x(1:2:end)); % Sum of even harmonics divided by sum of odd harmonics

HR_ML_d3 = sum(harmonics_3y(1:2:end)) / sum(harmonics_3y(2:2:end)); % Sum of odd harmonics divided by sum of even harmonics

HR_VT_d3 = sum(harmonics_3z(2:2:end)) / sum(harmonics_3z(1:2:end)); % Sum of even harmonics divided by sum of odd harmonics

% Visualize amplitudes of the first 20 harmonics

figure;

subplot(3, 1, 1);

bar(1:harmonics, harmonics_3x);

xlabel('D3 Sequence');

ylabel('Amplitude');

title('AP-axis First 20 Harmonics Amplitude');

subplot(3, 1, 2);

bar(1:harmonics, harmonics_3y);

xlabel('D3 Sequence');

ylabel('Amplitude');

title('ML-axis First 20 Harmonics Amplitude');

subplot(3, 1, 3);

bar(1:harmonics, harmonics_3z);

xlabel('D3 Sequence');

ylabel('Amplitude');

title('VT-axis First 20 Harmonics Amplitude');

% Store the current HR values

HRs_AP_d3(i) = HR_AP_d3;

HRs_ML_d3(i) = HR_ML_d3;

HRs_VT_d3(i) = HR_VT_d3;

% Update startRow and endRow for the next HR calculation

startRow = endRow + 1;

endRow = startRow + (peri * 2) - 1; % The length of the gait period is peri * 2

end

% Calculate the mean HR

mean_HR_AP_d3 = mean(HRs_AP_d3);

mean_HR_ML_d3 = mean(HRs_ML_d3);

mean_HR_VT_d3 = mean(HRs_VT_d3);

% Output results

fprintf('Mean HR\_AP\_d3 = %f\n', mean_HR_AP_d3); % Display gait harmonic ratio in the AP direction

fprintf('Mean HR\_ML\_d3 = %f\n', mean_HR_ML_d3); % Display gait harmonic ratio in the ML direction

fprintf('Mean HR\_VT\_d3 = %f\n', mean_HR_VT_d3); % Display gait harmonic ratio in the VT direction
