# Supplementary material for: Wearable accelerometers reveal objective assessment of walking symmetry and regularity in idiopathic scoliosis patients
Source: PeerJ. 2024 Jul 16;12:e17739. doi: 10.7717/peerj.17739 (PMC11259127; doi:10.7717/peerj.17739)
Supplement: Supplemental Information 4 [file peerj-12-17739-s004.doc]

STROBE Statement—checklist of items that should be included in reports of observational studies

|  | Item No | Recommendation |
| --- | --- | --- |
| **Title and abstract** | 1 | (*a*) Wearable Accelerometers Reveal Objective Assessment of Walking Symmetry and regularity in Idiopathic Scoliosis Patients |
|  |  | (*b*) Objective: This study investigates bilateral lower limb gait symmetry and regularity, along with the balance control strategy of head movements during walking in idiopathic scoliosis patients.  Design: 17 idiopathic scoliosis patients and 17 healthy subjects participated, with three-dimensional accelerometers were attached to their heads and L5 spinous process. Three-dimensional motion acceleration signals were collected during a 10-meter walking test. Analysis of the collected acceleration signals involved calculating five variables related to the symmetry and regularity of walking: root mean square (RMS) of the acceleration signal, harmonic ratio (HR), step regularity, stride regularity, and gait symmetry.  Results: Analysis reveals significant differences in lumbar region motion acceleration signals between idiopathic scoliosis patients and healthy controls, with lower RMS of the vertical axis (RMS-VT) (1.6±0.41 vs. 3±0.47, P＜0.05) and HR of the vertical axis (HR-VT) (3±0.72 vs. 3.9±0.71, P＜0.05). Head motion acceleration signals in three dimensions, including RMS, HR-VT, step regularity, and stride regularity, are significantly lower in idiopathic scoliosis patients compared to healthy controls (P＜0.05).  Conclusion: Idiopathic scoliosis patients exhibit distinctive irregularities in gait symmetry, particularly in the antero-posterior and vertical direction. The dynamic balance control strategy of their head movements in three-dimensional space is relatively conservative compared to healthy individuals. |
| Introduction | | |
| Background/rationale | 2 | Scoliosis is a three-dimensional deformity involving the skeletal and joint systems, typically accompanied by vertebral rotation in the transverse plane, alterations in the physiological curvature of the spine in the sagittal plane, and lateral curvature deformities in the coronal plane 1 . The Scoliosis Research Society (SRS) defines scoliosis as the measurement of the Cobb angle in the coronal plane X-ray imaging of the entire spine. If the Cobb angle is greater than 10 degrees and there is evident vertebral rotation, it is diagnosed as scoliosis 2 . Idiopathic scoliosis is a complex, multifactorial, and etiologically unclear neuro-musculo-skeletal disorder 3 .  Previous studies have indicated that patients with scoliosis experience impaired balance function, with asymmetry in the spine of scoliotic individuals not only altering the position of the center of mass in the trunk but also affecting the patient's balance control strategy4. Even in cases of mild spinal deformity (Cobb angle less than 25°), individuals with scoliosis demonstrate certain balance control impairments in simple postural control tasks 5. As the severity of spinal deformity increases, the impairment in balance control becomes more pronounced, and individuals tend to adopt a more conservative method of balance control 6. Current research attributes the insufficient postural balance control in individuals with scoliosis to two hypotheses: the biomechanical hypothesis and the sensory integration hypothesis 7. The former suggests that spatial displacement of the spinal deformity and the positions of the shoulders, pelvis, and head contribute to the decline in postural control. The latter proposes that inaccurate sensory input weights lead to compromised dynamic regulation of sensorimotor integration, causing a decline in balance control abilities.  Impairments in postural balance control capacity caused by idiopathic scoliosis directly influence the spatiotemporal parameters of patients' gait, resulting in abnormal gait patterns. Studies indicate that, compared to healthy subjects, individuals with idiopathic scoliosis exhibit a shorter stance phase and a longer swing phase 8. Correspondingly, pelvic joint activity and hip joint activity in the horizontal and sagittal planes during walking are reduced 4. However, previous research has primarily focused on the kinematics of the trunk and pelvis in scoliosis patients and changes in gait spatiotemporal parameters. Limited research exists on the symmetry of bilateral gait, and there is no consensus on the symmetry and regularity of bilateral lower limb gait 4. Moreover, most previous studies have analyzed gait spatiotemporal parameters and the motion of the trunk and pelvis to determine gait symmetry in individuals with scoliosis, using complex equipment such as plantar pressure plates or infrared three-dimensional motion capture systems6,9-11. However, direct measurements of gait symmetry and regularity were not commonly performed.  Spinal curvature in the sagittal, coronal, and horizontal planes alters the balance stability of patients in three-dimensional space. Neurosensory regulatory function maintains the trunk's static and dynamic stable upright posture, while abnormal head positioning affects both the head and trunk posture, ultimately leading to decreased trunk stability 12-14. Furthermore, maintaining head stability is essential for optimizing visual conditions, and maintaining head stability may be one of the fundamental tasks of the body posture control system during walking 15,16.  Studies have shown that, based on imaging measurements and pressure analysis of the plantar surface, patients with scoliosis and spinal imbalance exhibit greater head offset distances than scoliosis patients in a balanced group. Additionally, the percentage difference in load between left and right feet and between front and rear feet is greater than in the balanced group. The increased instability of the head and body during static standing exacerbates trunk imbalance 12. However, there is currently a lack of research on the three-dimensional dynamic balance control strategies of the head in scoliosis patients during walking and whether they affect the symmetry and regularity of bilateral walking. |
| Objectives | 3 | this study aims to comprehensively investigate the symmetry and regularity of bilateral lower limb gait in patients with scoliosis using data collected through three-dimensional accelerometers. Simultaneously, the study investigates the balance control strategies of the head during the walking process. Through this research, we hope to provide a convenient measurement method for assessing the abnormal gait in patients with scoliosis, and simultaneously offer new insights for future clinical practices. |
| Methods | | |
| Study design | 4 | A retrospective, single-center study |
| Setting | 5 | Idiopathic scoliosis subjects were recruited from December 2022 to June 2023. The subjects with normal spinal alignment and aged over 10 years as the control group through posters and online advertisements in the hospital. |
| Participants | 6 | Inclusion criteria were as follows: 1) Diagnosis by a rehabilitation doctor as Lenke type 1 (main thoracic curve) or Lenke type 5 (main lumbar curve) idiopathic scoliosis 17 ; 2) Age greater than 10 years; 3) Inclusion of idiopathic scoliosis patients with complete standing anteroposterior and lateral full-spine X-rays, with Cobb angles of the scoliotic curve greater than 10 degrees; 4) No history of wearing orthosis or surgery, no physical therapy or exercise training in the three months prior to inclusion; 5) Bilateral lower limb length difference within a range of 1 cm, capable of independent standing and walking; 6) Dominant foot on the left side; 7) Absence of flat feet. Exclusion criteria were: 1) Diagnosis of non-idiopathic scoliosis; 2) Severe deformities in the lower limbs and feet or deformities in other body parts; 3) Presence of other diseases affecting gait, such as trauma, muscle atrophy, or joint diseases related to musculoskeletal disorders.  The subjects with normal spinal alignment and aged over 10 years were recruited as the control group |
|  |  |  |
| Variables | 7 | The variables for each walking symmetry and regularity parameter are derived from calculations based on the X (AP), Y (ML), and Z (VT) axis data of the acceleration signal. The specific parameters include:  Acceleration Root Mean Square (RMS): RMS measures the dispersion of the measured acceleration signal relative to zero. It's a commonly used parameter to evaluate the dynamic stability of measured segments during walking, reflecting the fluctuation of measured segments during walking^18^.  Harmonic Ratio (HR): The HR represents the smoothness and rhythmicity of the acceleration pattern. The HR on the anterior-posterior axis (X) and the sagittal axis (Z) is calculated as the ratio of the sum of even harmonic values to the sum of odd harmonic values. While for the acceleration on the medial-lateral axis (Y), the HR is calculated as the ratio of the sum of odd harmonic values to the sum of even harmonic values, contributing to the assessment of acceleration in the medio-lateral direction^18^.  Step Regularity (AD1):AD1 represents the amplitude of the first peak in the acceleration autocorrelation signal.  Stride Regularity (AD2):AD2 represents the amplitude of the second peak in the acceleration autocorrelation signal.  Higher AD1 and AD2 values closer to 1 indicate better step regularity and stride regularity, respectively^19^.  Gait Symmetry: Gait symmetry is reflected by the closeness of AD1/AD2 to 1.0. A value closer to 1.0 indicates greater symmetry in gait^19^.  These parameters are indicative of various aspects of gait stability, smoothness, regularity, and symmetry and are calculated based on different aspects of the acceleration signal across multiple axes.  Walking Speed and cadence: The formula for calculating walking speed (V) is given by V=10/t，where t represents the time taken to walk 10 meters. The formula for calculating cadence (c) is c =60f/N, where f is the sampling frequency (60 Hz in this study), N is the number of samples per dominant period, and c is the cadence expressed in steps per minute(steps/min)^18^. |
| Data sources/ measurement | 8* | The experiment employed wearable three-dimensional acceleration sensors (Xsens DOT, Enschede, NL) (weight=11.2 g, dimensions =36.3 x 30.4 x 10.8 mm) to collect three-dimensional plane (sagittal, coronal, horizontal) motion acceleration signals separately at the head and lumber. The acceleration sensor had an internal sampling rate of 800 Hz, an output rate of 60 Hz, and a standard full range of ±16g. Dynamic mode was utilized for data collection, and the output acceleration signals were gravity-acceleration-removed. The lumber acceleration sensor was placed near the center of mass at the L5 spinous process using medical-grade double-sided tape and elastic bandage for fixation. The head sensor was positioned at the occipital pole using a headgear elastic band and medical-grade double-sided tape for fixation. The X-axis of the sensor pointed in the anatomical antero-posterior direction (AP), the Y-axis in the medial-lateral direction (ML), and the Z-axis in the vertical direction relative to the ground (VT).  During the 10-meter walking test, motion acceleration signals from the head and lumber of the subjects were simultaneously collected. Subjects were instructed not to turn their heads, keep their eyes straight ahead, and let their arms swing naturally. Upon receiving the start command, subjects walked forward in a natural state for 15 meters. Acceleration data for the 15-meter walk and the time taken by the subjects to traverse the central 10 meters were recorded. The test was repeated twice, and the mean of the two results was considered as the final measurement outcome. |
| Bias | 9 | To ensure data accuracy, it is imperative that a single individual conducts the data analysis. Throughout the data processing phase, the acceleration signals from the subjects were kept blinded to the data analysis researchers, and the data results were grouped only after the completion of the entire data analysis.  To mitigate potential bias, a single individual installed the acceleration sensors on both the head and lower lumber. Additionally, all subjects underwent the 10-meter walk test in the same location to ensure uniform testing conditions. |
| Study size | 10 | This study included a total of 17 patients with Lenke 1 and Lenke 5 types of idiopathic scoliosis, as well as 17 healthy subjects. |
| Quantitative variables | 11 | The acceleration signals from the subjects were kept blinded to the data analysis researchers, and the data results were grouped only after the completion of the entire data analysis. The raw acceleration data, from which the gravity component had been removed, underwent filtering and smoothing using MATLAB (R2014a, the MathWorks, Inc., Natick, MA, USA). A second-order Butterworth low-pass filter with a cutoff frequency of 10Hz was applied to ensure the representativeness and statistical significance of the results. Specifically, 420 points (7 seconds) from the middle of the acceleration signal were randomly selected as the data for calculating variables. Five variables reflecting walking symmetry and regularity were selected and calculated in MATLAB. |
| Statistical methods | 12 | Statistical analysis of experimental data was performed using SPSS 20.0 (IBM, Chicago, IL, USA). Measurement results were expressed as mean ± standard deviation. Initially, the Kolmogorov-Smirnov normality test was conducted. For data conforming to normal distribution, an independent t-test was employed to analyze the differences in walking symmetry and regularity variables between the two groups of subjects. For data that did not pass the normality test, the Mann-Whitney U test was applied as a non-parametric test. A significance level of P < 0.05 was considered statistically significant. |
|  |  |  |
|  |  |  |
|  |  |  |
|  |  |  |

Continued on next page

| Results | | |
| --- | --- | --- |
| Participants | 13* | This study included a total of 17 patients with Lenke 1 and Lenke 5 types of idiopathic scoliosis, as well as 17 healthy subjects. |
|  |
|  |
| Descriptive data | 14* | (a) The demographic characteristics of the two groups, including age, weight, and gender ratio, were similar (p > 0.05) (Table 1). Among the 17 included patients with idiopathic scoliosis, 6 were classified as Lenke 1 type, and 11 as Lenke 5 type. Each idiopathic scoliosis subject provided complete standing anteroposterior and lateral full-spine X-rays, with an average Cobb angle of 21.75±6.56 for the main curve. |
|  |
|  |
| Outcome data | 15* | Root mean square (RMS) of the acceleration signal, harmonic ratio (HR), step regularity, stride regularity, gait symmetry, walking speed and cadence |
|  |
|  |
| Main results | 16 | **Walking symmetry and regularity variables**  The analysis results of walking symmetry and regularity variables show significant changes in the measurements of the lumbar and head regions for subjects with idiopathic scoliosis compared to healthy subjects (Table 2*)*.  In comparison to healthy individuals, patients with idiopathic scoliosis exhibit a decrease in RMS in both the lumbar and head regions across all three directions, but only the RMS in the VT exhibits a significant difference (p < 0.05). For the RMS on the ML, there is no significant difference in the head and lumbar regions between the idiopathic scoliosis group and the healthy control group (p > 0.05). In the AP direction, although the head RMS of the idiopathic scoliosis group is significantly lower than that of the healthy control group (0.49±0.22 vs. 0.65±0.22 respectively, p＜0.05), there is no significant difference between the two groups in the lumbar region (p＞0.05) (Fig 2). Regarding the HR, the overall HR values of idiopathic scoliosis subjects are smaller than those of the healthy control group, but statistical differences are only present in the VT direction (p＜0.05) (Fig3).  When evaluating the regularity of gait, the step regularity of the head in idiopathic scoliosis significantly decreases in both the AP and VT directions (p < 0.05) (Fig 4). Additionally, the stride regularity in all three directions of the head is significantly lower than that of the healthy control group (p < 0.05) (Fig 5). However, for the lumbar region, both step regularity and stride regularity in all three directions show no significant difference compared to the healthy control group (p＞0.05).  The gait symmetry in the head and lumbar regions between the idiopathic scoliosis group and the healthy control group does not exhibit significant differences across all three directions (p > 0.05). Additionally, no significant distinctions were found in terms of 10-meter walking speed and cadence when comparing the two groups (p>0.05) (Table 2). |
|  |
|  |
| Other analyses | 17 |  |
| Discussion | | |
| Key results | 18 | Analysis reveals significant differences in lumbar region motion acceleration signals between idiopathic scoliosis patients and healthy controls, with lower RMS of the vertical axis (RMS-VT) (1.6±0.41 vs. 3±0.47, P＜0.05) and HR of the vertical axis (HR-VT) (3±0.72 vs. 3.9±0.71, P＜0.05). Head motion acceleration signals in three dimensions, including RMS, HR-VT, step regularity, and stride regularity, are significantly lower in idiopathic scoliosis patients compared to healthy controls (P＜0.05). |
| Limitations | 19 | The primary concern centers on the representativeness of the study sample. Exclusively comprising patients diagnosed with idiopathic scoliosis of Lenke 1 and 5, the sample entails a highly specific selection of research subjects. The average Cobb angle among individuals with scoliosis is a modest 21.75±6.56, indicative of mild spinal curvature. This particularity in participant selection may potentially constrain the generalizability and representativeness of the research findings. A further consideration revolves around constraints associated with the employed technological apparatus. Despite the use of three-dimensional accelerometers as measurement instruments, this technology exhibits certain limitations. Specifically, accelerometers might fall short in furnishing intricate biomechanical insights, encompassing joint angles and muscle activity, potentially constraining a holistic comprehension of gait abnormalities. In subsequent investigations, it becomes imperative to systematically address these constraints, thereby augmenting the scientific rigor and trustworthiness of the study. |
| Interpretation | 20 | The investigation revealed that individuals with idiopathic scoliosis exhibit abnormalities in the symmetry and regularity of their gait during the walking process, discernible across the AP, ML, and VT direction. Furthermore, there is a relatively conservative dynamic balance control strategy for the head in three-dimensional space. |
| Generalisability | 21 | The utilization of wearable three-dimensional accelerometers sensors presents a promising and convenient approach to assess the symmetry and regularity of walking in patients with idiopathic scoliosis. These research findings contribute novel insights into the gait symmetry, regularity, and measurement for individuals with idiopathic scoliosis, offering valuable references for prospective diagnostic. Subsequent studies should further investigate the correlation between lower limb gait asymmetry, spinal deformity progression, and relevant mechanical functional characteristics in patients with idiopathic scoliosis. |
| Other information | | |
| Funding | 22 | This work was supported by our institution. |

*Give information separately for cases and controls in case-control studies and, if applicable, for exposed and unexposed groups in cohort and cross-sectional studies.

**0**
